# High-Strength and Low-Cost Biobased Polyurethane Foam Composites Enhanced by Poplar Wood Powder Liquefaction

**DOI:** 10.3390/polym13172999

**Published:** 2021-09-04

**Authors:** Wanjia Yang, Yanming Han, Wei Zhang, Derong Zhang

**Affiliations:** 1 Research Institute of Wood Industry, Chinese Academy of Forestry, Xiangshan Road, Beijing 100091, China; yangwanjia@bjfu.edu.cn; 2 Key Laboratory of Wood-Based Materials Science and Utilization, Beijing Forestry University, No. 35 Tsinghua East Road, Haidian District, Beijing 100083, China; zhdr666666@sina.com.cn; 3 Beijing Key Laboratory of Wood Science and Engineering, Beijing Forestry University, No. 35 Tsinghua East Road, Haidian District, Beijing 100083, China

**Keywords:** polyurethane foam composites, poplar wood powder liquefaction, liquefaction, environmentally friendly

## Abstract

An environmentally friendly liquefaction of wood powder was prepared by atmospheric pressure liquefaction technology to replace the non-renewable petroleum polyols in the preparation of polyurethane foam composites. The liquefaction time varied from 0 min to 140 min. The composition of liquefied products and the effects of liquefaction time on the morphology, apparent density and mechanical properties of polyurethane foam composites were investigated. The results showed that the optimal process time for the preparation of wood powder liquefaction products, which could replace traditional petroleum polyols, was 110 min. At this time, polyether polyols are the main liquefaction products, with an average molecular weight in *Mn* reaching 237 and average molecular weight in *Mw* reaching 246. The functional group of the liquefied product consisted mainly of hydroxyl, with the highest content of 1042 mg KOH/g and the lowest acid number of 1.6 mg KOH/g. In addition, the surface of the polyurethane foam based on poplar wood is dominated by closed cell foam; thus its foam has good heat insulation and heat preservation properties. At 110 min liquefaction time, the apparent density of polyurethane foam is 0.164 g/cm^3^ and the compression strength is 850 kPa, which is higher than that of traditional polyurethane foam (768 kPa), which is without wood powder modification. Replacing petroleum polyol with renewable wood powder liquefaction products to prepare biomass-based polyurethane foam composite materials, researching complex chemical changes in different liquefaction stages, and finding the best liquefaction conditions are of great significance to optimize the performance of polyurethane, address the shortage of resources and reduce environmental pollution.

## 1. Introduction

Polyurethane foam composites are one of the most popular polymeric materials. They are polymers prepared by the reaction of polyether polyols or polyester polyols with binary isocyanate or polyisocyanates, with carbamate (-NH-COO-) repeating structural units. They have a wide range of applications in construction, refrigeration, and insulation, among others, because of their good electrical conductivity, low thermal conductivity, mechanical properties, and good shock absorption.

Polyester polyols and polyether polyols are important chemical intermediates in the production of polyurethane materials. However, at present, the raw materials of polyester and polyether polyols consist of toxic and corrosive petrochemical products, such as phthalic anhydride and phthalic acid. The use of such non-renewable oil resources has resulted in pollution and threats to the natural environment. The most effective way to address this issue is to replace polyester polyols or polyether polyols with renewable woody biological raw materials to prepare green, environmentally friendly and biodegradable biomass-based materials, such as biomass-based polyurethane foam [1].

Lignocellulose includes lignin, cellulose and hemicellulose as well as a small amount of organic matter such as fat, protein, pectin and ash. Lignocellulosic biomass is not only a renewable, abundant and cheap resource with a large amount of hydroxyl groups, but also has a stable three-dimensional network structure, which can replace and modify petroleum polyols in chemical production. Lima García et al. [2] found that high purity lignin dispersed to sufficiently small particle size can improve the lap shear strength and the wood failure percentage of bonded beech specimens as well as the gap filling properties of resin. Manggar et al. [3] successfully prepared high strength, low curing temperature, short pressing time, and isocyanate-free lignin-based non-isocyanate polyurethane resins. Tavares et al. [4] prepared polyurethanes based on renewable raw materials (technical Kraft lignin, castor oil and modified castor oil), and the results indicated that the starting materials change the polymer’s properties. The oil modification process improved hydroxyl concentration and led to polyurethanes with enhanced mechanical properties versus ones synthesized from unmodified castor oil. The products show the feasibility of developing polyurethane-type materials with large property ranges, by using an industrial, low-cost, unmodified and largely available residue combined with a non-edible renewable source oil. Ravindra et al. [5] successfully used lignins as part of the polyol to synthesize lignin-polyurethane-based wood adhesives. A slower setting time and better adhesion to the wood substrate was observed as compared to the standard PU, and the toxicity was also reduced. These results support the development of polyurethane adhesive using lignin as a natural and renewable polyol, allowing the reuse of this industrial waste. Lignocellulose fiber has low density, high specific strength and stiffness, good heat insulation and sound insulation performance; it is widely used in the toughening modification of various materials. Poplar is a common fast-growing tree species in China because of its light weight and fast growth rate. Poplar fiber has been widely used in composite materials because of its low cost, high yield, easy processing, high elasticity, long fibers and high content. Therefore, in this study, wood fibers of white poplar were used to toughen polyurethane foams [6]. Lignocellulose, as a green, natural material, not only can reduce the use of petroleum polyols, but also can give polyurethane materials biodegradable performance, reduce the secondary pollution of products, and bring new hope for the development of the polyurethane industry.

At present, the application of lignocellulose in polyurethane materials includes two main aspects: on the one hand, lignocellulose is directly used as a compatibilizer or filler material; on the other hand, lignocellulose is chemically modified or liquefied to produce polyols as the raw material for polyurethane preparation [7]. The composition of lignocellulose is complex, and the structure and physicochemical properties of its three-dimensional network polymers are different due to the different sources and separation methods. Therefore, it is difficult to directly prepare polyurethane materials using lignocellulose as a compatibilizer or filler [8]. Compared with chemical modification, liquefaction is simpler and also one of the most effective methods for resource utilization. Liquefaction under atmospheric pressure, in particular, has attracted much attention because of its mild reaction conditions and remarkable liquefaction effect [9]. Liquefaction technology can convert the chemical composition of biomass into a liquid polymer material, which can be used in the production of adhesives and foams [10]. It is a thermochemical process of converting wood biomaterials directly from solid state to liquid state with the help of a liquefied solvent under certain temperature and pressure conditions.

By means of liquefaction, natural polymers such as cellulose, hemicellulose and lignin in lignocellulose can be degraded into liquid products with relatively low molecular weight and certain reactivity. These liquefied products can be further used as fuel or chemical raw materials [11]. The liquefaction of woody biomass under atmospheric pressure can be realized by selecting proper solvents as liquefaction reagents. Compared with high-temperature and high-pressure liquefaction methods, liquefaction under atmospheric pressure is a mild liquefaction method [8].

In practical application, different liquefaction solvents are often chosen according to the final use of liquefied products. If polyhydroxy alcohols, such as ethylene glycol (EG), polyethylene glycol (PEG) or a mixture of the two solvents, are used as liquefaction solvents, the liquefaction products are mainly used to prepare polyurethane adhesives or polyurethane foams. If phenol is used as the liquefaction solvent, its liquefaction products are usually used to prepare thermoplastic and thermosetting phenolic resins, foaming materials, molded materials, carbon fibers, etc. [12].

Polyethylene glycol (PEG) is usually used as the liquefaction reagent in the liquefaction of lignocellulosic biomass. Because polyethylene glycol itself can be polymerized with an isocyanate monomer, the liquefied products of lignin biomass can be directly used in the preparation of polyurethane materials without any separation. When polyethylene glycol is used as a liquefaction reagent, it is usually necessary to add a small amount of small molecule polyhydroxyl compounds (ethylene glycol, glycerol, etc.) as an auxiliary liquefaction reagent to inhibit the condensation reaction [8]. Kurimoto et al. found that the addition of 11.1% glycerol to polyethylene glycol could reduce the residue rate and delay the occurrence of polycondensation reaction, the addition of 33.3% glycerol could inhibit the occurrence of polycondensation reaction, and the addition of glycerol improved the availability of wood liquefaction products [13].

When powdered lignocellulosic macromolecules are liquefied to obtain reactive small molecules, which are then added to the preparation of polyurethane foam, the compatibility between liquefied products and isocyanates is increased, and the properties of polyurethane are also improved. However, due to the structure and morphology of different biomasses, a large number of complex chemical reactions take place simultaneously and compete with each other in the liquefaction process of wood powder [14]. Shiraishi et al. [15] used hydroxy-propylated wood solution to prepare polyurethane foam. The results showed that although the foam had high strength and good compressive deformation recovery ability, its apparent density was as low as 0.04 g/cm^3^ and thus its mechanical properties were insufficient. Consequently, the liquefaction condition of wood powder is an important element. The liquefaction of wood with polyether polyols or polyester polyols may further improve the properties of the foamed plastics. As a result, it is of great practical significance to study the complex chemical changes in different stages of wood powder liquefaction and find the optimal liquefaction conditions for the preparation of wood-powder-based polyurethane foam, which can replace petroleum polyols with biomass energy, optimize polyurethane properties and reduce costs.

In this paper, the liquefaction mechanism of different lignocellulose components under different liquefaction times as well as the effects of different liquefaction times on the structure, surface morphology, apparent density and compressive strength of polyurethane foam were studied.

## 2. Materials and Methods

NaOH (98%), glycerol, polyethylene glycol 400 (PEG400, average molecular weight of 400), concentrated sulfuric acid (mass fraction 98%), phthalic anhydride and pyridine were provided by Lan-yi Chemical Products Co. Ltd., Beijing, China.

The wood powder of white poplar (40 mesh) was provided by Chengzun Mineral Products Processing limited company, Ling-shou County, Shijiazhuang, China.

The following were provided by Bailing Qingyue Polyurethane Company, Beijing, China: diphenylmethylene diisocyanate (MDI), industrial grade, isocyanate content of 30.31%; polyether polyols 4110 and polyether polyols 403, industrial grade; triethylenediamine solution (A33), industrial grade; dimethyl methyl phosphate (DMMP), industrial grade; silicone oil, industrial grade; 1, 1-Dichloro-1-fluoroethane (HCFC-141B), industrial grade. 

### 2.1. Preparation of Poplar-Powder-Based Polyols

#### 2.1.1. Instruments and Equipment

The collection type constant temperature heating magnetic agitator DF-101S, Bangxi Instrument Technology (Shanghai, China) Co., LTD, was used to stir the sample solution evenly.

A multi-function electric stirrer, RCT B S025, IKA, Germany, was used to stir the wood powder and liquefy the solvent when liquefying the wood powder.

A vacuum drying oven, DZF-6053, Yi-heng Scientific Instrument Co., Ltd., Shanghai, China, was used to mature the polyurethane foam.

#### 2.1.2. Methods

A solvent mixture of poplar wood powder, PEG400 and glycerol and catalyst sulfuric acid were added in proportion to a separable flask containing an agitator, thermometer and reflux condenser. Then, the separable flask was dipped into a pre-heated oil bath and stirred for a period of time. After the reaction, the separation flask was immediately placed in a beaker with ice water to cool down and stop the reaction. NaOH was added to the flask and the value of pH of the solution was adjusted to 7 and let stand overnight. The residue was filtered out, and the supernatant was poplar-powder-based polyols.

This experiment used PEG/glycerol 4:1 (W/W), liquid-solid ratio 9:1, liquefaction temperature 130 °C, sulfuric acid (catalyst) dosage 1.8% (sulfuric acid in the mass ratio of liquefaction solvent and wood powder raw material), liquefied for 20 min, 50 min, 80 min, 110 min, 140 min.

#### 2.1.3. Determination of Hydroxyl Value of Liquefied Products

The hydroxyl value of the liquefied product was determined with reference to GB/T12008.3-2009 [16], “Plastic polyether polyols—Part 3: Determination of hydroxyl value”. The principle method used was as follows: the hydroxyl in the sample is esterified by reflux in pyridine solution of phthalic anhydride; imidazole is used as the catalyst; the excessive anhydride is hydrolyzed in water; the phthalic acid generated is titrated with sodium hydroxide standard titration solution; and the hydroxyl value is calculated by the difference between the sample and blank titration.

The phthalic anhydride acylation reagent was prepared as follows: weave 116 g of phthalic anhydride and dissolve in a 1 L brown bottle; add 700 mL of pyridine and vigorously shake until dissolved; add 16 g of imidazole and carefully shake until dissolved. The solution should be left to rest overnight before use.

The methods used were as follows: Sample 1 to 1.5 g with a syringe into a conical flask, and add 25 mL of phthalic anhydride acylating agent to each sample and blank conical flask using a pipette. Shake the flask until the test material is dissolved. Connect each conical flask with an air-condensing tube and place in an oil bath at (115 ± 2) °C for reflux for 30 min; shake the flask 1~2 times during reflux. After heating, remove the device from the oil bath and cool to room temperature. Rinse the condensing tube drop by drop with 30 mL pyridine and remove the condensing tube. Quantitatively transfer the solution to a 250 mL beaker and rinse the flask with 20 mL pyridine. Stir the beaker on a magnetic stirrer and determine the hydroxyl value of the liquefied product by potentiometric titration. Titrate the solution with a standard titration solution of 0.5 mol/L NaOH to pH = 7. Conduct the blank test in the same way. Calculate the hydroxyl value (*OHV*) according to Equation (1):

(1) $$OHV=\frac{(V_{4}-V_{3})\cdot c\times 56.1}{m}$$

- *V*_3_: the volume of the NaOH standard titration solution consumed when titrating the sample, mL;
- *V*_4_: the volume of the NaOH standard titration solution consumed when titrating the blank, mL;
- *c*: the concentration of the NaOH standard titration solution, mol/L;
- *m*: quality of test material, g;
- 56.1: molar mass of KOH, g/mol.

#### 2.1.4. Determination of Acid Number of Liquefied Products

The acid number of the liquefied product was determined by referring to GB/T12008.5-2010 [17], “Plastics polyether polyols—Part 5: Method for determination of acid value”. The principle method used was as follows: dissolve the sample in isopropyl alcohol, make phenolphthalein an indicator, and add 0.02 mol/L potassium hydroxide methanol standard solution to the terminal point at room temperature.

The conical flask was filled with (100 ± 20) mL isopropyl alcohol and 1 mL phenolphthalein indicator solution. The solution was titrated to light pink with 0.02 mol/L potassium hydroxide methanol standard solution for 30 s. A total of 50~60 g of test material was deposited into the conical flask and the quality of the test material was recorded, accurate to 0.1 g. The solution in the conical flask was shaken until the sample was completely dissolved. The sample solution was titrated with 0.02 mol/L potassium hydroxide-methanol standard solution. The end point was determined by potentiometric titration, and the volume consumed was recorded.

The acid value C of the sample was measured by the amount of KOH consumed, in mg/g, and calculated according to Equation (2):

C = (A × N × 56.1)/W(2)

A—volume of potassium hydroxide-methanol standard titration solution consumed by titration test material, mL;

N—concentration of potassium hydroxide-methanol standard titration solution, mol/L;

56.1—molar mass of KOH, g/mol;

W—quality of test material, g.

#### 2.1.5. Determination of Relative Molecular Weight and Distribution of Liquefied Products

The relative molecular weight and distribution of the liquefied products removed from the residue were determined using a 1525 high-performance liquid chromatograph, Waters Company, US, and PL-GPC220 high-temperature gel penetration chromatograph, Agilent Company, US. The analysis conditions were as follows: the mobile phase was tetrahydrofuran, the flow rate was 1 mL/min, the detector temperature was 30 °C, the standard sample was polystyrene and the molecular weight range was 500–3,000,000.

#### 2.1.6. Fourier Transform Infrared Spectroscopy (FT-IR) Spectroscopic Evaluations of Liquefied Products

The Nicolet 6700 Fourier transform infrared spectrometer from American Nicolet Instrument Company was used. The determination resolution was 4 cm^−1^, the number of scans was 64 and the spectral range was 400–4000 cm^−1^.

#### 2.1.7. Nuclear Magnetic Resonance (NMR) Spectroscopy of Liquefied Products

The ASCEND 400 liquid NMR instrument, Bruker, Germany, was used under the following 13C-NMR measurement conditions. The solvent was pyridine, the frequency was 150.92 MHz, the number of scanning times was 6144, the pulse angle was 90°, the pulse width was 4.50 μs, the capture time was 0.56 s and the delay time was 2 s. Total test time was 13 h.

### 2.2. Preparation and Characterization of Polyurethane Foam Composites Based on Liquefied Poplar Wood Powder 

#### 2.2.1. Material Calculation in the Preparation Process of Liquefied Modified Poplar-Based Polyurethane Foaming Material

The chain expansion, foaming, crosslinking and other reactions occurring in the preparation of polyurethane foaming materials all involve the material ratio, and the correct calculation of material ratio is key to ensure the synthesis of polyurethane foaming materials. If the amount of isocyanate is too small, that is, it is lower than the theoretical calculation value, it will cause insufficient growth of the polymer chain or cause an insufficient amount of CO_2_ produced by reaction with the water, which will directly affect the mechanical properties and density of the foam. If the amount of isocyanate is too large, it will not only lead to the wasting of raw materials but also lead to a fast reaction rate and fast chain growth rate, resulting in the formation of too many crosslinking bonds, such as biuret and urea-formate, which will seriously affect the balance between the foaming rate and gel rate, resulting in the phenomenon of brittle and hard foams [18].

The type and dosage of each component in the formula were determined according to the literature as well as development and production experience. As shown in Table 1.

After determining the dosage of polyols, catalysts, foaming agents and foaming stabilizers, the dosage of isocyanate components was calculated. The amount of isocyanate used in this study refers to the total amount of isocyanate required in the one-step synthesis process of polyols and water. The amount of isocyanate is calculated using Equation (3).

(3) [NCO]/[OH]=MMDI×WMDIMLL×WLL+Mpolyol ×Wpolyol+(218)×Wwater

- *M_MDI_*: number of substances containing the isocyanate group per gram of *MDI*, mmol/g;
- *W_MDI_*: mass of isocyanate *MDI*, g;
- *M_LL_*: amount of hydroxyl in the liquefied product per gram, mmol/g;
- *W_LL_*: mass of the liquefied product, g;
- *M_polyol_*: amount of hydroxyl in polyether polyols per gram, mmol/g;
- *W_polyol_*: quality of polyether polyols, g;
- *W_water_*: quality of the water, g.

The total amount of isocyanate used in the foaming formula, in addition to the amount required in the above formula, should also consider the degree and purity of the excess isocyanate used in the foaming process. Therefore, the amount of isocyanate used in the foaming formulation is generally greater than that required in Equation (3).

#### 2.2.2. Synthesis Formula and Preparation Method of Liquefied Modified Poplar-Based Polyurethane Foaming Material

Polyether polyols 4110 (40 g) and 403 (60 g) were added into the paper cup at a certain ratio, and a quantitative amount of poplar-based polyols was added. The polyols were dried in a vacuum drying oven at 70 °C for 10 min. The catalyst triethylene diamine (A33) and dibutyltin dilaurate, foam stabilizer silicone oil, flame retardant dimethyl methyl phosphate (DMMP), foaming agent 1,1-dichloro-1-fluorine ethane (HCF-141B) and a small amount of water were successively added. The isocyanate was added and stirred vigorously for 10~15 s under an electric stirrer at 3000 r/min to stir the material evenly, so that chain growth, gas generation and cross-linking reactions could be carried out almost simultaneously in a short time. Then, the mixture was quickly poured into the preheated mold, allowing free expansion and foaming for 1 min at room temperature. After the foam stopped growing, it was placed in an oven at 65 °C for 3 h to mature. Finally, the foam was placed at room temperature for 24 h until it was fully cured, and the properties of the polyurethane foaming materials were characterized. The ambient temperature was 10~20 °C, and the ambient humidity was 50~70%.

#### 2.2.3. Observation of the Pore Morphology of Polyurethane Foam Materials

Scanning electron microscopy (SEM) 7610F and 7900 from Nippon Electronics Corporation were used. Before observation, the sample was placed on the sample table for gold-plating treatment. The gold-plated sample was placed under 10 kV acceleration voltage for observation, and the shooting was magnified 30 times.

#### 2.2.4. Determination of Density of Polyurethane Foaming Materials

Part of the properties of polyurethane foams can be described by the apparent density. The apparent density of the foam material was measured according to GB/T6343-2009 [19]. The sample size was 30 mm × 30 mm × 30 mm, and the original bubble structure should not be destroyed when cutting. Five samples of each polyurethane foam material were made, and their mass was measured and recorded in g. The specific calculation method is as follows: take the average value of the five samples as the apparent density of polyurethane foam material, accurate to 0.1 kg /m^3^.

(4) $$\rho =\;\frac{m}{v}\times 10^{3}$$

- *ρ*—performance density (g/cm^3^)
- *m*—foam material quality (g)
- *v*—foam volume (mm^3^)

#### 2.2.5. Determination of Compression Properties of Polyurethane Foam Composites

A universal mechanical testing machine (Instron 3366, Instron, Boston, MA, USA) was used for measurement at room temperature according to ASTM D1621—16 standard [20]. The sample size was 30 mm × 30 mm × 30 mm, and the compression speed was 5 mm/min. The compression strength and compression modulus of the material were read and recorded. Each group was repeated 5 times, and the average value was taken. Tests were conducted in the standard laboratory atmosphere of 23 °C ambient temperature and 50% relative humidity.

## 3. Results and Discussion

### 3.1. Characterization of Liquefied Products

#### 3.1.1. Fourier Transform Infrared Spectroscopy (FT-IR) Analysis of Liquefied Products of Poplar Powder

**Figure 1:**
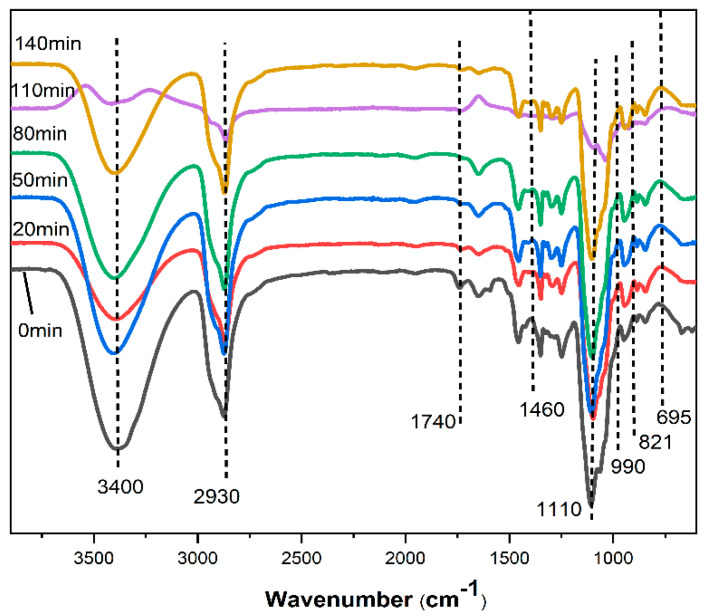
FT-IR of liquefied products at different liquefaction times.

As shown in Figure 1, the wide absorption peak at 3400 cm^−1^ is the alcohol hydroxyl of the polyols or the stretching vibration peak of the phenol hydroxyl and the -OH of the alcohol hydroxyl in the chain segment of the degradation products of poplar wood powder. With the extension of liquefaction time, the peak wave number of hydroxyl (-OH) stretching vibration of the liquefied product is the lowest and the peak shape is the widest when the liquefaction time is 110 min, which may be due to the existence of hydrogen bond association in the liquefaction system before 110 min, resulting in the increase of unsaturated bonds in the liquefied products. At the same time, the content of hydroxyl in the liquefied product increased, indicating that the functional group of the liquefied product was dominated by hydroxyl. At 110 min, the peak type of hydroxyl stretching vibration was the widest and the wave number was the lowest. FTIR spectra do not describe hydroxyl groups quantitatively, only qualitatively. Thus, the FTIR spectra of 110 min liquefaction product showed the lowest wave peak, which only indicates that the content of hydroxyl increased and a large number of hydrogen bond associations existed. 

With the increase of liquefaction time, the content of the hydroxyl group changed constantly, which is due to the complexity of the reaction in the liquefaction process of wood powder. With the change of time, the dominant chemical reaction in each stage is different. Because of the different structure and morphology of different biomasses, a large number of chemical reactions take place simultaneously and compete with each other in different stages of liquefaction. This is caused mainly by the different liquefaction rates, due to the different structures of cellulose, hemicellulose and lignin [9]. Typically, liquefaction of hemicellulose, lignin, and amorphous cellulose occurs rapidly in the early stages of the liquefaction process, due to their amorphous structure and easy contact with the liquefaction solvent. On the contrary, crystallized cellulose, due to its dense structure, is not easily in contact with the liquefied solvent, and its liquefaction rate is usually slow and continues until the later stage of the liquefaction process [21,22,23].

**Figure 2:**
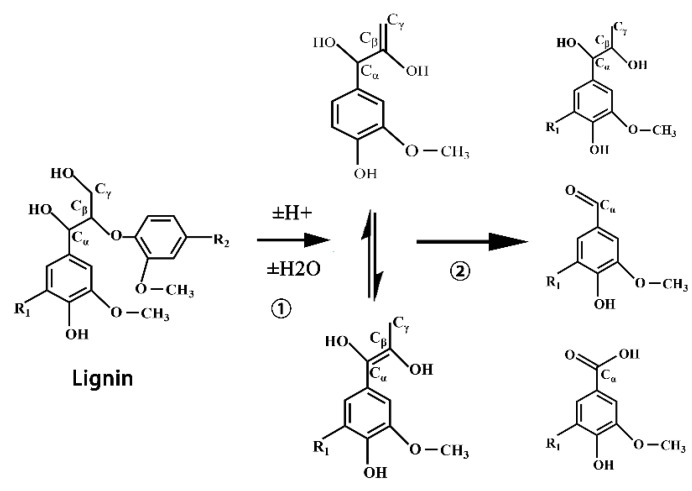
The degradation reaction (①) and polycondensation (②) of lignin.

**Figure 3:**
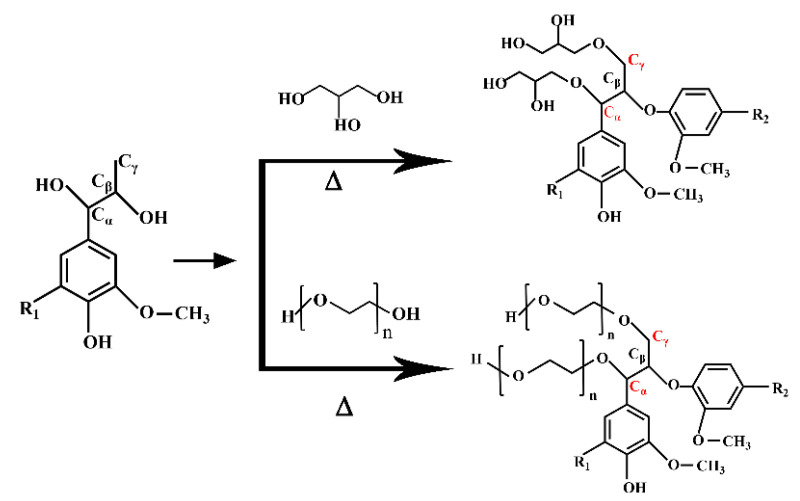
Reaction of lignin degradation products with liquefied solvents to form polyols.

At 2930 cm^−1^ and 1460 cm^−1^, the stretching and bending vibrations of C-H in methylene were respectively observed. The strength of these two peaks first increased, then decreased, and then increased. The peaks of 2930 cm^−1^ and 1460 cm^−1^ were the stretching and bending vibration of C-H in methylene, respectively. The strength of these two peaks first increased, then decreased and then increased. This enhancement may be due to the cracking of lignocellulose, as shown in Figure 2, or the phenol hydroxyl group of phenylpropane structure of poplar powder participating in the liquefaction reaction, as shown in Figure 3. At this time, the small solvent molecules were immersed into the macromolecular structure of poplar powder through alkylation reaction. Its weakening may be due to the polycondensation of lignin. Most of the increased methylene is due to the introduction of PEG400 into the lignocellulosic structure, as shown in Figure 3.

The strong absorption peak of C=O at 1740 cm^−1^ indicates the ester bond between hemicellulose and lignin in wood powder or the acylation phenomenon in wood powder. However, the strength of the absorption peak decreased or even disappeared in the products after liquefaction, which indicates that the units connected by ester bonds may have broken under the liquefaction condition and each component of wood powder was degraded. There was an absorption peak at 1642 cm^−1^, which is the C=O stretching vibration absorption peak of carbonyl group in penta-hemiacetal, indicating that there are aldehydes in the liquefied product. The peak of 1600 cm^−1^ belongs to the characteristic peak of benzene ring structures, indicating that the liquefied product contains a benzene ring structure, which comes mainly from the lignocellulose of poplar wood powder and its derivatives. At 1500 cm^−1^ is the C=C stretching vibration absorption peak on the aromatic framework of the benzene ring. The results show that the liquefying made the components in wood solvate with the liquefying agent and produce more aromatic derivatives. The bending vibration of C-H in cellulose was 1371 cm^−1^. The in-plane bending vibration peak of the phenolic hydroxyl group was 1340 cm^−1^, indicating the formation of phenolic compounds.

**Figure 4:**
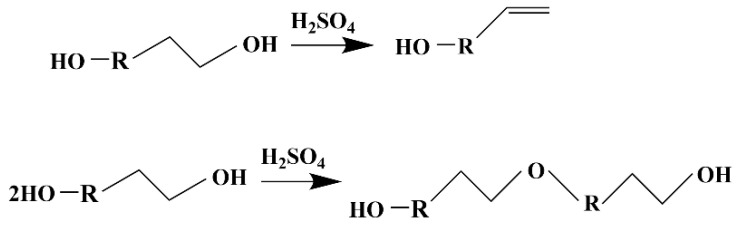
Condensation reaction between liquid solvents of polyols.

The stretching vibration of C-O in lignin was 1264 cm^−1^, and the asymmetric stretching vibration of C-O-C in cellulose was 1162 cm^−1^. The absorption peak at 1110 cm^−1^ was caused by C-O-C symmetric stretching vibration, indicating that there are ether compounds in the liquefied products, which may come from the degradation of lignin, as shown in Figure 2, or the condensation reaction between liquefied solvents, as shown in Figure 4.

The furan ring skeleton vibration at 990 cm^−1^ may be the product of cellulose degradation by PEG 400. The 942 cm^−1^ characteristic peak is attributed to the out-of-plane bending vibration of the C-H bond on the poly-substituted benzene ring. At 895 cm^−1^, the characteristic poly-substituted peak of the benzene ring was observed, which indicates that a hydroxyl-methylation reaction occurred between the liquefied solvent and phenolic substances in wood powder, and the reactivity of the liquefied solvent was enhanced. The strongest absorption peak at 821~695 cm^−1^ is the C-H bond vibration of aromatic hydrocarbons, which indicates that compounds with different substituents are generated on the benzene ring, more groups are exposed, and the activity of the benzene ring is enhanced.

In summary, the main functional group of wood powder liquefaction products is hydroxyl. The liquefied product is a mixture of polyether polyols, aromatic compounds, phenols, aldehydes and ethers. At the same time, in different stages of wood powder liquefaction, there is lignocellulose degradation reaction, lignin polycondensation reaction, the condensation reaction between the liquefied solvent itself, the alkylation reaction of small molecules of the solvent, as well as the hydroxy-methylation reaction between the liquefied solvent and phenolic substances in the wood powder and other complex reactions. 

#### 3.1.2. Nuclear Magnetic Resonance (^13^C-NMR) of Poplar Powder and Liquefaction Products 

**Figure 5:**
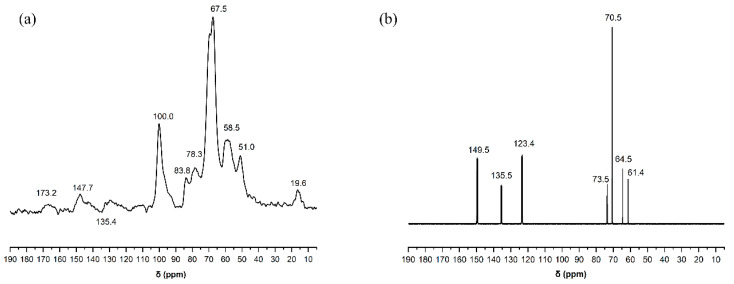
(**a**) ^13^C-NMR spectra of poplar powder. (**b**) ^13^C-NMR spectra of 110 min liquefaction products.

In order to understand the structure of liquefied products clearly, ^13^C-NMR was used to characterize poplar wood powder and its polyhydroxy alcohol liquefaction products. Figure 5a,b list the ^13^C-NMR spectra of poplar powder and liquefaction products with a liquefaction time of 110 min, respectively.

In the spectrogram of wood powder, the peak of the chemical shift in the range of 110~160 ppm was attributed to the carbon atoms on the benzene ring, and the peak type was more complex, which was related to the lignin in the poplar powder containing more benzene ring connections. The peaks of chemical shift at 147.7 ppm and 135.4 ppm were attributed to the C-4 atom in the guaiacol benzene ring and C-1 atom in the syringa-β-O-4 structure, respectively. These phenyl ring signal peaks were strongly enhanced in the spectra of liquefied products, indicating that the phenyl ring structures were separated from the wood powder after liquefaction. In addition, the peak of un-etherized C-4 atoms of lignin at 147.7 ppm became narrower and were concentrated at 149.5 ppm, which is attributed to etherized C-4 atoms, indicating that lignin hydrolyzed to produce small molecules with ether bonds.

The peak of the liquefied product at 123.4 ppm belongs to the carbon atom in the structure of para-hydroxy-phenylpropane, while the wood powder does not have this peak, which also indicates that the wood powder was degraded into small molecules. 

In the spectrogram of wood powder, the chemical shift peak at 100 ppm belongs to the C-2 and C-6 atoms of phenolic syringyl. These signal peaks disappeared in the spectra of the liquefied products, indicating that the benzene ring structure of lignin molecules was modified by polyhydroxyl alcohol liquefaction under acidic conditions, and then hydroxyl groups were added to the benzene ring. The signal peak of chemical shift at 83.8 ppm belongs to the C-4 atom in the amorphous zone of cellulose and C_β_ atom in the monomer structure of lignin, and the signal peak at 78.3 ppm belongs to the C-2, C-3 and C-5 atoms in the crystalline zone of cellulose and C_α_ atom in the monomer structure of lignin. 

**Figure 6:**
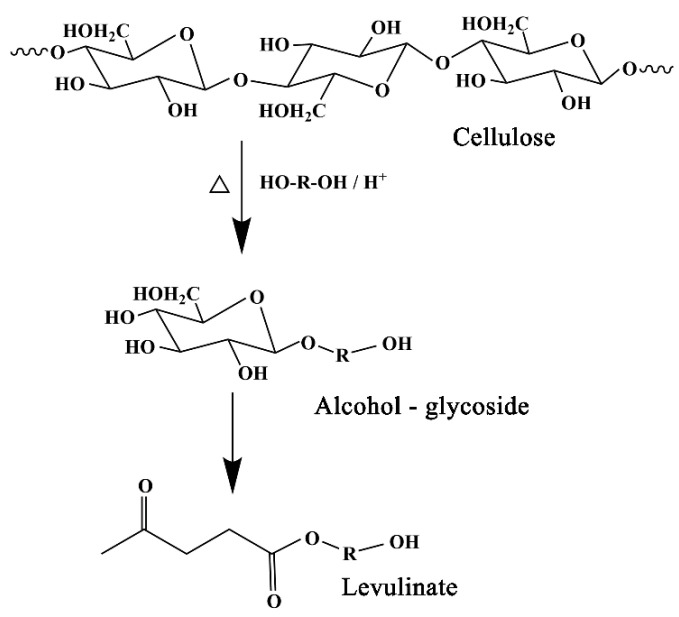
Macromolecular cellulose degradation into small fatty alcohol glycosides.

Tatsuhiko Yamada and Hirokuni Ono [24] analyzed the composition of the ethylene glycol (EG)-liquefied products as a function of liquefaction time, and the findings indicate that the degradation of cellulose during ethylene glycol (EG) liquefaction has the following mechanism: first, cellulose is degraded and produces considerable ethylene glycol (EG)-glucosides during the early stage of liquefaction. Second, when liquefaction is prolonged, glucosides are decomposed, leading to a large quantity of levulinates. This mechanism is illustrated in Figure 6. In the spectra of liquefied products, the signal peaks at 83.8 ppm and 78.3 ppm disappeared, indicating that the crystalline region of cellulose was broken, macromolecular cellulose degraded into small fatty alcohol glycosides, and the small molecular aliphatic chain of the liquefied reagent entered the monomer structure of lignin through addition reaction. 

Compared with the spectra of wood powder, the peak of liquefied products varied significantly between 75 and 60 ppm, which indicates that the small molecules of liquefied solvent were involved in the degradation reaction of wood powder, and the lignin degradation products of wood powder and the solvent had alkylation reaction. The strong signal peak at 58.5 ppm and 51.0 ppm of chemical shift was attributed to the methoxy group (-OCH_3_), connected by the side chain of the syringa ring and guaiacol benzene ring. In the spectra of liquefied products, the methoxy signal peaks disappeared at 58.5 ppm and 51.0 ppm, indicating that demethylation reaction occurred in the syringes and the side chains of the guaiacyl benzene rings in lignin. The strong signal peaks with chemical shifts at 173.2 ppm and 19.6 ppm were attributed to the acetyl carboxyl and acetyl methyl groups of hemicellulose, respectively, which were degraded by Arabinoxylan, arabinan, and galacto-glucomannan [25]. In the spectra of liquefied products, the signal peaks at 173.2 ppm and 19.6 ppm disappeared, indicating that hemicellulose was involved in the liquefaction reaction. And when the liquefaction time was 110 min, the hemicellulose had been degraded.

It can be seen from the above analysis results that the original chemical structure of poplar wood powder changes to some extent when it is liquefied by poly-hydroxyl alcohol under acidic conditions, and new compounds are formed. Moreover, there are many types of liquefied products, which are relatively complex. The active intermediates, formed in the process of liquefaction, can be aliphatic alcohols, guaiacol and other compounds, which may further be condensed with solvent small polyols or with each other to produce condensates with relatively large molecular weights such as polyether polyols, resulting in a difference between the liquefaction product structure and the structure of the wood powder.

#### 3.1.3. Hydroxyl Value and Acid Number of Liquefied Products

**Figure 7:**
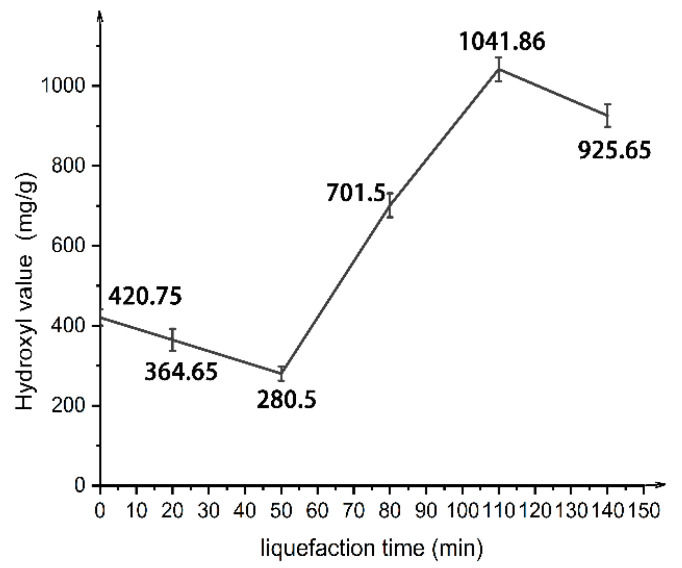
Hydroxyl value of liquefied products at different liquefaction times.

As can be seen from Figure 7, with the liquefaction time from 0 to 110 min, the hydroxyl value of the wood powder liquefaction product first decreased and then increased, reaching a maximum value of 1042 mg KOH/g at 110 min. This is because, before 50 min, the liquefaction time is short, and the liquefaction reaction is dominated by the degradation of lignin [26,27], resulting in the generation of intermediates with active points [28]. Thus, the number of hydroxyl groups decreases and the hydroxyl value decreases.

However, between 50 min and 110 min, with the increase of reaction time, the intermediate after lignin degradation reacts with solvents such as polyethylene glycol 400 or polyols to form relatively stable substances, and hydroxyl terminations and sub-hydroxyl groups are introduced [28]. Therefore, when aliphatic hydroxyl groups enter the mixed liquefaction system, the hydroxyl value of the liquefied product increases, which is consistent with the results shown by the infrared spectra and ^13^C NMR spectra of the liquefied product. Moreover, due to the dense structure of crystalline cellulose, it is not easy to make contact with the liquefied solvent, so the liquefaction rate is usually slow and will continue until the later stage of the liquefaction process. Therefore, at this time, a large amount of cellulose is likely to begin to degrade into fatty alcohol glycosides, and the crystallization zone is broken, exposing more reactive hydroxyl groups. This is consistent with the change of -OH wide absorption peak at 3400 cm^−1^ of the liquefied product in the infrared spectrum. 

When the liquefaction time was 110 min, the hydroxyl value reached the maximum. Meanwhile, the infrared spectrum of the liquefied products also showed that, at this time, the peak type of the stretching vibration of the hydroxyl group was the widest and the wave number was the lowest. The FTIR spectra above do not describe hydroxyl groups quantitatively, only qualitatively. Thus, the FTIR spectra of the 110 min liquefaction product showed the lowest wave peak, which only indicates that the content of hydroxyl increased and a large number of hydrogen bond associations existed. The hydroxyl values can be quantitatively expressed by the hydroxyl value graph. The hydroxyl value diagram showed that the value of the 110 min liquefaction product was the highest. Therefore, the results of the FTIR spectra and the hydroxyl value do not conflict, and the best liquefaction time of poplar powder is 110 min.

When the liquefaction time exceeded 110 min, the hydroxyl value of the liquefied product decreased again. This may be due to the polycondensation of lignin, or it may be due to the polycondensation of polyethylene glycol 400 itself, resulting in a decrease in the hydroxyl value. Meanwhile, fatty glycosides formed by cellulose degradation and liquefied solvent are further degraded to levulinic acid [29], resulting in the reduction of hydroxyl groups in the liquefied system. In addition, polyhydroxy alcohols, the liquefaction agent, are also prone to intramolecular and intermolecular dehydration reactions under acidic high temperature conditions and exist in the liquefied products in the form of ether bonds.

**Figure 8:**
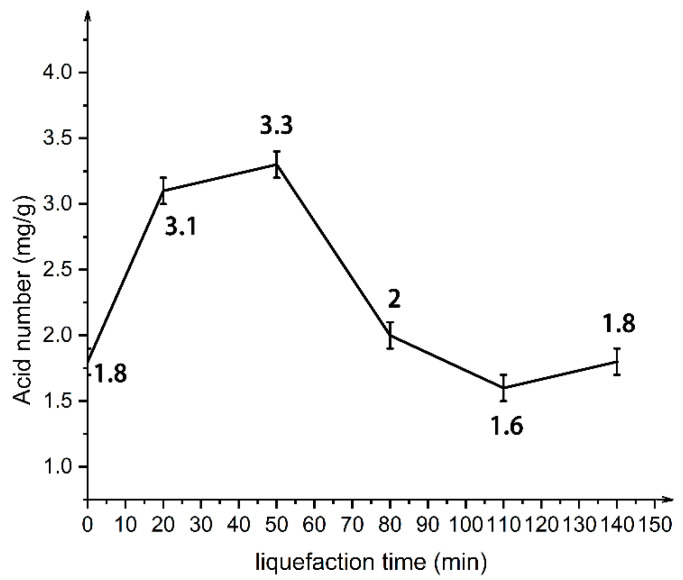
Acid number of liquefied products at different liquefaction times.

Figure 8 shows the acid number of liquefied products at different liquefaction times. With the increase of liquefaction time, the acid number of liquefied productions increases first, then decreases, and then increases. Between 0–50 min, the reason for the increase of the acid value may be the oxidation reaction of polyols heated in sulfuric acid to generate carboxylic acid, with the liquefaction product containing the free carboxylic group. As the temperature continues to rise, between 50 min and 110 min, the carboxylic acid generated reacts with polyols and enzymatic lignin degradation molecules to form new ester bonds, so the acid value shows a downward trend. After 110 min, the acid value increases, possibly because at this time, the fatty alcohol glycosides from the cellulose degradation and liquefaction solvent are further degraded into levulinic acid [29].

In short, wood powder polyols liquefaction is a complicated process, including the biomass degradation reaction of polymer and liquefied reagent hydroxy alcohol, the solvent reaction between low molecular compounds, the condensation reaction between the generation of low molecular compound condensation and self-condensation, and the oxidation of liquefacient condensation reaction. The main functional group of the liquefied product was hydroxyl. The optimal liquefaction time was 110 min, and the hydroxyl value reached 1042 mg KOH/g.

#### 3.1.4. Gel Penetration Chromatography (GPC)

The identification and separation of small molecules of a substance can be achieved by gel penetration chromatography (GPC) and can also be used to analyze the chemical properties of molecular weight homologues of different polymers. By means of gel permeation chromatography, the molecular weight of the liquefied product and the degree of molecular chain destruction can be detected to determine the type of the liquefied product.

**Figure 9:**
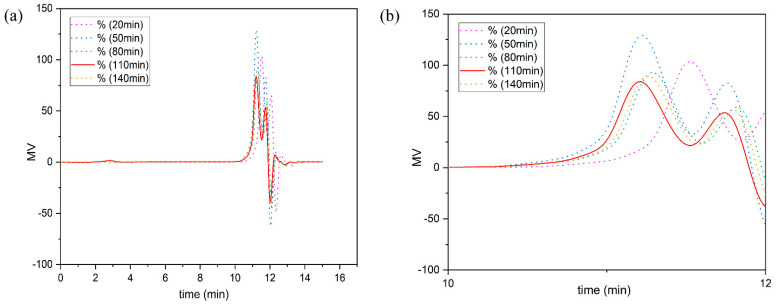
(**a**) GPC spectra of liquefied products at different liquefaction times. (**b**) Local enlarged view of GPC spectra of liquefied products at different liquefaction times.

In the determination of the relative molecular weight, the liquefied product used is the liquefied product after the removal of the residue. Figure 8 shows the relative molecular weight distribution of liquefied products at different liquefaction times (20 min, 50 min, 80 min, 110 min, and 140 min). The ordinate (MV) represents the refractive index difference between the sample and the solvent, and the response signal of the differential refractive detector is proportional to the concentration of the solute. The abscissa represents the retention time. As shown in Figure 9a, GPC curves of liquefaction products with different liquefaction times include two main parts: the part with retention time between 10 and 11 min and the part near 11.5 min. According to the principle of gel permeation chromatography, the former peak on the GPC spectrum represents the compound with large molecular weight, and the latter peak represents the compound with small molecular weight.

**Figure 10:**
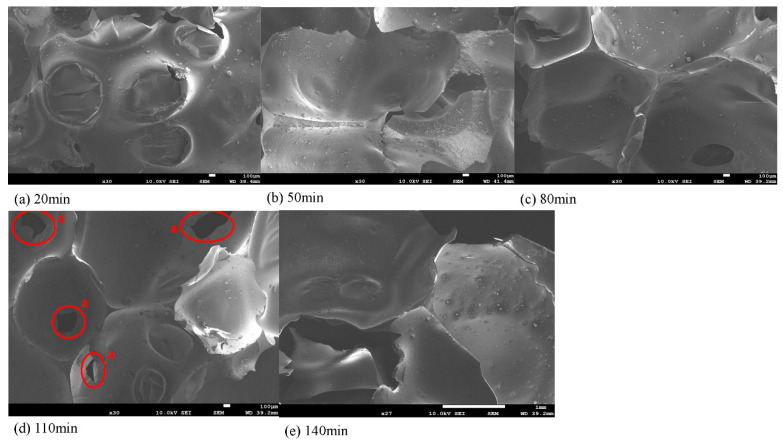
SEM micrographs of polyurethane foams prepared using different polyols at different liquefaction times.

Figure 9b is a GPC enlarged view of liquefaction products with different liquefaction times (residence time from 8 min to 12 min). Figure 10 shows the GPC curve of liquefaction products with a liquefaction time of 20 min, 50 min, 80 min, 110 min and 140 min. The former peak represents the liquefied products of wood flour with polyhydroxy alcohol. The latter peak is a solvent peak, and represents the molecular fragments of liquefied reagents with low relative molecular weight; the solvent peak is not related to molecular weight but to reagent and instrument state. Thus, the former peak is the focus of this paper and is also the basis for the calculation of the number average molecular weight and weight average molecular weight of the liquefied product. The separation between the former peak and the latter peak is obvious, so it will not interfere with the relative molecular weight of the liquefied product.

The relative molecular weights (wide peaks) of the liquefied products at different liquefaction times are listed in Table 2. It can be seen from Table 2 that the average molecular weight of the liquefied products of wood powder first increases and then decreases. This indicates that after the poplar wood powder is liquefied by the polyhydroxy alcohol solvent, the alcoholysis reaction takes place, and the lignin macromolecules are degraded into smaller molecules. When the liquefaction time exceeded 20 min, the relative molecular weights of *Mn*, *Mw* and *Mp* of the liquefied products showed an increasing trend. This indicates that after the poplar wood powder is liquefied by polyhydroxy alcohol solvent, the alcoholysis reaction takes place, and the lignin macromolecules are degraded into smaller molecules. Then, the intermediate after lignin degradation reacts with solvents such as phenol or polyol to form a more stable substance. The molecular weight decreased from 50 min to 80 min, probably because a significant amount of cellulose began to degrade into small molecules. The increase in molecular weight from 80 min to 110 min may be due to the polycondensation reaction between lignin derivative molecules or the polyethylene glycol itself, which generates condensates with relatively large molecular weight, or the degradation of cellulose into fatty alcohol glycosides [1].

Min N et al. [30] explored the polycondensation reaction mechanism of wood powder in the liquefaction process of polyols and also found that within the liquefaction time of 0–180 min, the polycondensation reaction occurred about 70 min later. The decrease from 110 min to 140 min may be due to the further degradation of fatty glycosides into levulinic acid [29]. Fangeng Chen et al. [31] found that after the liquefaction of wheat straw with ethylene glycol, the GPC analysis of the liquefied products showed that with the progress of the liquefaction reaction, the contents of both high molecular weight and low molecular weight products increased gradually, indicating that condensation and degradation of ethylene glycol in the liquefaction process of wheat straw existed simultaneously. Lee et al. [32] liquefied wood powder with phenol as a liquefaction agent under supercritical conditions and analyzed the liquefied products with GPC. It was found that the molecular weight and its distribution decreased significantly at the initial stage of the liquefaction reaction and further decreased with the slight increase of the reaction time. This indicates that wood powder is liquefied and degraded into small molecules, but as the reaction time continues to extend, the small molecules produced by liquefaction undergo condensation polymerization to produce macromolecules again, resulting in an increase in molecular weight.

In conclusion, the optimal liquefaction time of poplar wood powder is 110 min. At this time, the liquefied product is mainly a mixture of polyether-polyols, with a number average molecular weight of *Mn* reaching 237 and a weight average molecular weight of *Mw* reaching 246. The liquefaction of wood flour with polyhydroxyl alcohol is a complicated process, which includes degradation reaction, solvation reaction and polycondensation reaction, and the degradation reaction and polycondensation reaction are a pair of competitive reactions. Therefore, there are many kinds of liquefied products with complex structures. Our exploration and analysis of the product structure was limited to the existing conditions.

### 3.2. Characterization of Polyurethane Foam

#### 3.2.1. Scanning Electron Microscopy (SEM)

Figure 10 shows the effect of liquefaction time on the micromorphology of the foaming pores of the cross section of lignin-based polyurethane foaming material. In Figure 9, the lignin-based polyurethane foaming materials prepared by liquefaction products of different liquefaction time are listed under the scanning electron microscope (SEM), which is 300 times larger.

As can be seen from Figure 10, there are a large number of granular substances on the surface of the foam holes of the polyurethane foaming material prepared by the liquefaction product with the liquefaction time of 20 min, which may come from lignocellulosic that does not participate in the cross-linking reaction. At this time, the foam hole shape of the foaming material is incomplete, which also indicates that the reaction between the liquefied product and isocyanate is not complete.

However, compared with the liquefaction products prepared at other times, the surface particles of the polyurethane foaming material prepared at the liquefaction time of 110 min were the least, the closed cell rate of the bubble pores was the highest, and the bubble morphology was the most complete, which indicates that the wood powder has a large number of hydroxyl compounds after a period of liquefaction modification, which is conducive to the cross-linking reaction with isocyanate.

**Figure 11:**
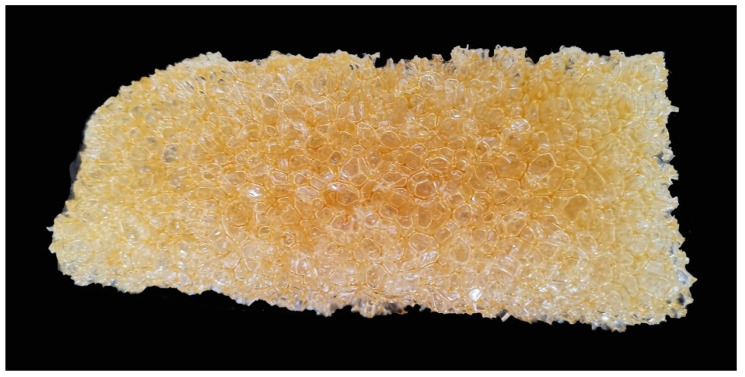
Cross-sectional view of polyurethane foams prepared using liquefied products at 110 min liquefaction time.

Figure 11 illustrates the cross-sectional view of polyurethane foams prepared using liquefied products at 110 min liquefaction time; the average diameter of the circular pores is about 2 mm. As can be seen from the figure, the bubble pores at this time are uniform and small, which is similar to the shape of polyurethane bubbles in previous studies [32].

#### 3.2.2. Apparent Density of Polyurethane Foaming Materials

**Figure 12:**
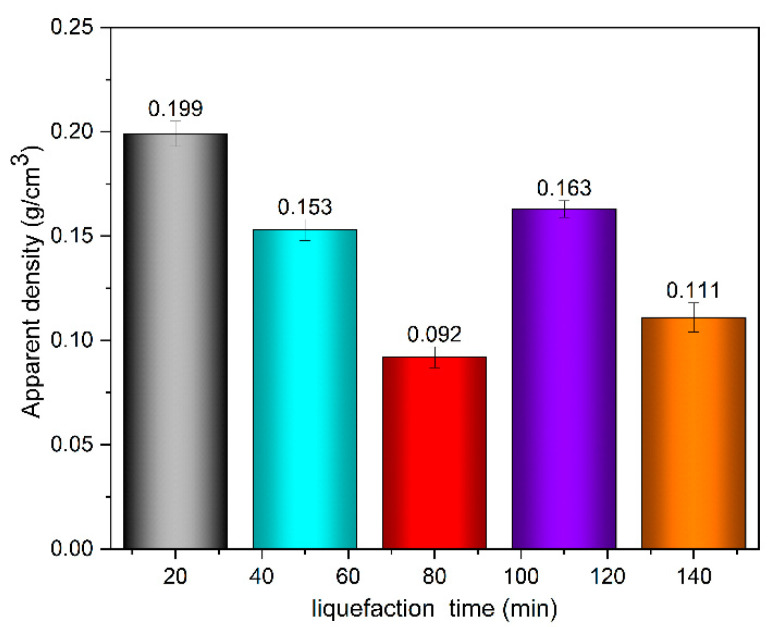
Effect of liquefaction time on the apparent density of polyurethane foaming materials.

Figure 12 shows the effect of liquefaction time on the apparent density of wood-powder-based polyurethane foaming material. It can be seen that with the extension of liquefaction time, the density of polyurethane foaming material decreases first, then increases, and then decreases. Therefore, the density change of polyurethane foaming material is probably related to hydroxyl value.

When the liquefaction time increased from 20 min to 80 min, the density of wood powder based polyurethane foaming material decreased from 0.199 g/cm^3^ to 0.092 g/cm^3^. This may be because a large amount of lignin degrades into reactive intermediates at the early stage of liquefaction, while a small amount of fatty alcohol glycosides degrades, and liquefaction reagent self-polymerization and other complex reactions occur. These reactions make the foaming system become a multiphase system, and the viscosity of the system increases, which is not conducive to the cross-linking reaction. In addition, the gas produced during foaming is difficult to overflow, so that the density is slightly reduced.

With the increase of liquefaction time from 80 min to 110 min, the density of polyurethane foaming material increased. This may be because, with the increase of reaction time, the intermediate after lignin degradation reacts with solvents such as polyethylene glycol 400 or polyols, introducing terminated hydroxyl groups and sub-hydroxyl groups [33]. Therefore, aliphatic hydroxyl groups enter the mixed liquefaction system, and the hydroxyl value of the liquefied product increases. In addition, at the same time, the crystallization zone of a large amount of cellulose is broken, and a large number of compounds with active hydroxyl groups are exposed; a large number of liquefied products subsequently react with isocyanate to form a stable foam structure. Therefore, the density of polyurethane foam increases. The bubble is difficult to expand, and the phenomenon of coalescence of bubbles is reduced, so that the aperture of the material and the density decreases.

The foam density of 0.163 g/cm^3^ at 110 min is larger than the highest polyurethane foam density of 0.110 g/cm^3^ prepared by previous studies [32], indicating that the polyurethane foam prepared in this study has small pore size and stable foam structure.

When the liquefaction time continued to increase to more than 110 min, the hydroxyl value of the liquefied products decreased, so that the liquefied products that reacted with the isocyanate decreased and the density of the polyurethane foaming material decreased.

#### 3.2.3. Compressive Stress and Compressive Strength

**Figure 13:**
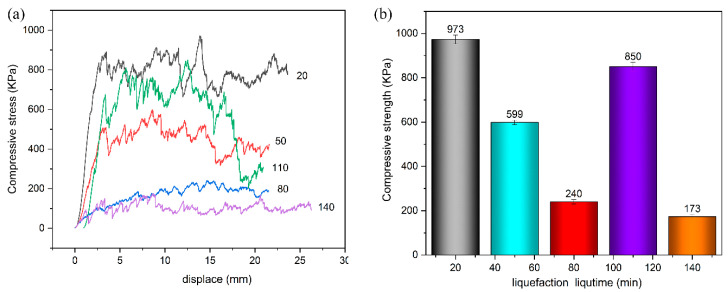
(**a**) The influence of liquefaction time on the compressive stress of polyurethane foaming materials. (**b**) The influence of liquefaction time on the compressive strength of polyurethane foaming materials.

The influence of liquefaction time on the compressive stress and compressive of wood powder based polyurethane foaming materials is listed in Figure 13a,b, respectively. With the prolongation of liquefaction time, the compression stress and compressive strength of polyurethane foaming materials change in the same trend, which decreases first, then increases, and then decreases. This is also consistent with the hydroxyl value and the density of the foaming material changing with the liquefaction time. Therefore, the change of compression stress and compression strength of polyurethane foaming material is likely related to hydroxyl value.

According to the FTIR spectrum and hydroxyl value diagram, the wood flour was not degraded at 20 min, nor did it react with chemical polyols to form wood flour polyols. Thus, most 20 min polyurethane foams are prepared by the reaction of chemical polyols (i.e., PEG400, glycerol) with isocyanates. The reaction between the wood flour polyol and isocyanate begins after 20 min.

When the liquefaction time increased from 20 min to 80 min, the compression stress and compressive strength of polyurethane foam material decreased. This may be because the lignin degradation products have the three-dimensional network structure of phenylpropane, which makes the liquefied products react with isocyanates to form a more complex cross-linking network. The rigid structure leads to brittleness of the foam, resulting in a decrease in strength. 

With the increase of liquefaction time from 80 min to 110 min, the compression strength of polyurethane foaming material increases. This indicates that with the increase of hydroxyl value, more liquefied products react with isocyanates to form a more complex cross-linking network, and the compression strength also increases. 

When the liquefaction time of wood powder is 110 min, the compression strength of wood powdered polyurethane foaming material reaches the maximum value of 850 kPa, which is higher than the compression strength of traditional polyurethane foam without wood powder at 768 kPa [32], so it has good mechanical properties. However, the compression strength of the polyurethane foam was slightly lower than the compression strength of 973 kPa of the wood-powdered polyurethane foaming material with the liquefaction time of 20 min. This may be because wood flour itself is soft and has a small amount of air left inside. During the formation of the bubble structure, a small amount of air in the wood powder will break through the weak part of the foam material and gather together, causing tiny cracks in the sample and increasing internal stress, as shown in Figure 10a–d. Therefore, when a large amount of wood powder enters the polyurethane foam system, the compression strength of the rigid polyurethane foam decreases slightly.

As the liquefaction time continues to increase, the compression strength of polyurethane foam decreases. This may be because when the liquefaction time exceeds 110 min, the hydroxyl value of the liquefied products decreases, so the liquefied products reacting with isocyanate decrease, and thus the compression strength of the polyurethane foaming material decreases.

## 4. Conclusions

In this paper, by measuring the hydroxyl value of wood powder liquefaction products with different liquefaction time, infrared spectra, GPC spectra and 13C-NMR spectra, it was found that with the liquefaction time from 0 to 110 min, the hydroxyl value of wood powder liquefaction products first decreased and then increased. This is mainly because the polyol liquefaction of wood flour is a complex process, and different components of wood flour react differently at different stages. Moreover, it is also accompanied by the degradation reaction of biomass macromolecules, the solvation reaction between the liquefaction reagent polyhydroxy alcohol and the generated low molecular compound, as well as the condensation reaction between the generated low molecular compound (auto-condensation reaction) and the oxidation condensation reaction of the liquefaction agent. The hydroxyl value of the wood powder liquefaction product reached the maximum value of 1042 mg KOH/g when the liquefaction time was 110 min. Therefore, the optimal process time for the preparation of the wood powder liquefaction product, which could replace the traditional petroleum polyols, was 110 min. In addition, through the characterization of the morphology, apparent density and mechanical properties of polyurethane foam prepared by liquefaction products of wood powder with different liquefaction times, it was found that the polyurethane foam prepared by liquefaction products at 110 min had the smoothest surface, and the compression strength of 850 kPa was higher than that of 768 kPa of traditional polyurethane foam without wood powder; thus, the mechanical properties are good.

## Figures and Tables

**Table 1:** Formulation of liquefied wood-powder-based polyurethane foaming material.

Component	Mass Percent %	Roles
Wood-powder-based polyols (20 g)	10–70	
polyether polyol 4110	12–54	cross-linking agent
polyether polyol 403	18–36	
silicone oil	3	foam stabilizer
water	2	chemical foaming agent
1,1-dichloro-1-fluorine ethane	15–25	physical foaming agent
triethylene diamine	1–4	catalyst
dibutyltin dilaurate	1–4	catalyst
dimethyl methyl phosphate	20–30	fire retardant

Note: The sum of the mass percentage of the liquefied product of polyols and enzymatic hydrolysis lignin is 100.

**Table 2:** The relative molecular weights of the liquefied products at different liquefaction times.

Sample	Number-Average Molecular Weight (*Mn*)	Weight-Average Molecular Weight (*Mw*)	Peak Molecular Weight (*Mp*)	Polydispersity
20 min	144	149	145	1.04
50 min	232	241	233	1.04
80 min	209	218	210	1.04
110 min	237	246	238	1.04
140 min	219	228	220	1.04

## Data Availability

This study did not report any data.
